# The Unresponsive Partner: Roles of Social Status, Auditory Feedback, and Animacy in Coordination of Joint Music Performance

**DOI:** 10.3389/fpsyg.2017.00149

**Published:** 2017-02-14

**Authors:** Alexander P. Demos, Daniel J. Carter, Marcelo M. Wanderley, Caroline Palmer

**Affiliations:** ^1^Department of Psychology, McGill UniversityMontreal, QC, Canada; ^2^Department of Music Research, CIRMMT, McGill UniversityMontreal, QC, Canada

**Keywords:** joint action, temporal coordination, social status, dynamical systems, auditory feedback

## Abstract

We examined temporal synchronization in joint music performance to determine how social status, auditory feedback, and animacy influence interpersonal coordination. A partner’s coordination can be bidirectional (partners adapt to the actions of one another) or unidirectional (one partner adapts). According to the dynamical systems framework, bidirectional coordination should be the optimal (preferred) state during live performance. To test this, 24 skilled pianists each performed with a confederate while their coordination was measured by the asynchrony in their tone onsets. To promote social balance, half of the participants were told the confederate was a fellow participant – an equal social status. To promote social imbalance, the other half was told the confederate was an experimenter – an unequal social status. In all conditions, the confederate’s arm and finger movements were occluded from the participant’s view to allow manipulation of animacy of the confederate’s performances (live or recorded). Unbeknownst to the participants, half of the confederate’s performances were replaced with pre-recordings, forcing the participant into unidirectional coordination during performance. The other half of the confederate’s performances were live, which permitted bidirectional coordination between performers. In a final manipulation, both performers heard the auditory feedback from one or both of the performers’ parts removed at unpredictable times to disrupt their performance. Consistently larger asynchronies were observed in performances of unidirectional (recorded) than bidirectional (live) performances across all conditions. Participants who were told the confederate was an experimenter reported their synchrony as more successful than when the partner was introduced as a fellow participant. Finally, asynchronies increased as auditory feedback was removed; removal of the confederate’s part hurt coordination more than removal of the participant’s part in live performances. Consistent with the assumption that bidirectional coupling yields optimal coordination, an unresponsive partner requires the other member to do all the adapting for the pair to stay together.

## Introduction

When musicians perform together, they must coordinate and adapt their actions in different social contexts. A musical ensemble, for example, can have a hierarchy with a principal director (such as a conductor of an orchestra) and sub-directors (such as the first violinist), or they may have a more equal or egalitarian relationship among members, as seen in some string quartets (Gilboa and Tal-Shmotkin, 2012). Regardless of the social context, the musicians must stay in tight temporal coordination to have a successful performance. To achieve this coordination, musicians rely on the auditory feedback from their own actions and the sound of their partners’ actions to adapt to and anticipate each other (Goebl and Palmer, 2009; van der Steen and Keller, 2013). The success of synchronization between performing musicians may also depend on the directionality of influence, referred to as coupling in dynamical systems theory; for example, one performer may influence the other (unidirectional coupling), or both may influence each other (bidirectional coupling). In order to contrast the types of coupling, we test the synchronization between pairs of pianists while we manipulate the social relationships between the partners, the access to their auditory feedback, and the direction of influence between the partners.

A non-linear dynamical systems perspective can explain the synchronization between two people in terms of *coupling*, or an energy transfer, that facilities the adjustment of their actions to maintain a stable phase relationship (Haken et al., 1985; Kelso, 1997; Pikovsky et al., 2001; Strogatz, 2003; Marsh, 2010, 2013; Latash, 2014). An energy transfer between two people typically occurs through perceptual information, such as when people use auditory feedback about their partners’ actions to adjust their actions (Néda et al., 2000; Riley et al., 2011; Demos et al., 2012). Coupling between people can be unidirectional or bidirectional. In unidirectional coupling, one system adapts to changes in the phase or period of the second system, such as a pianists adapting to a recording. Bidirectional coupling occurs when both systems respond and adapt to one another (Pikovsky et al., 2001), such as two pianists adapting to each other. Current dynamical mathematical models suggest that bidirectional coupling yields an optimal form of coordination as each person can share in the adapting (Strogatz, 2000), whereas unidirectional coupling would require all of the adaptation to occur by one member of the pair to maintain synchrony. Temporal coordination in joint music performance may be unidirectional (as when a performer plays with a non-responsive recording) and would be expected to generate less synchrony or bidirectional (as when a performer plays with a responsive live partner) and would be expected to generate more synchrony. We compare live and recorded performances in a manipulation of duet performance, in which the participants do not know whether the confederate’s performance is animate (live) or not (recorded).

Marvel et al. (2009) describe the shift of social relationships in a group of people as arising from inequalities in energy transfer among the members. Originating from applications of balanced relations in graph theory (Cartwright and Harary, 1956), Marvel et al. (2009) interpret the connections in a social network as an energy minimization process. This theory defines an energy landscape with certain relationships within the social network as more stable than others, with the intrinsic goal to avoid unbalanced (unstable) relationships. One can apply this concept to a musical relationship with groups as small as two; for example, musical duets composed of equally or unequally experienced or informed members. In our design, we manipulate how much knowledge the participant believes the confederate has about the task. We create either a balanced relationship in which the confederate is an equal partner in the task, or an unbalanced relationship in which the confederate is an experimenter in the task. The latter instruction is designed to suggest the confederate has more knowledge, power, experience, or information about the task, and thus the social relationship is imbalanced. Although a social imbalance may affect the way performers perceive each other, we do not expect it to drastically affect the degree of temporal coordination, as social imbalance during music performance is relatively common; for example, when one ensemble member is in charge of directing the group, all ensemble performers must stay coordinated in time or else the music will not sound correct.

Auditory feedback during joint music performance can also create imbalance among musicians. Studies of auditory feedback effects have generally used one of two manipulations: those that manipulate the feedback from live performance, and those that manipulate the effects of recorded performance feedback. Studies that manipulate feedback from live performance suggest that the removal of self-feedback causes less disruption to temporal coordination than the removal of the partner’s feedback (Goebl and Palmer, 2009; Loehr and Palmer, 2011; Zamm et al., 2015). Those studies also show that the more auditory feedback that is removed, the larger the asynchrony becomes between duet performers. Studies that manipulate feedback from performers playing with audio recordings suggest that performers can synchronize better with recordings of their own performances than with recordings of other performers (Keller et al., 2007). As well, studies suggest that there are individual differences related to a performer’s ability to synchronize with a recorded partner (Novembre et al., 2013). To our knowledge, no study has yet compared directly the effects of auditory feedback on adaptation to synchronization between live and recorded performance, one goal of the current study.

We examined the temporal synchronization between duet pianists while the social relationship of the pair was manipulated. Each participant pianist was introduced to their partner as either an experimenter (imbalanced hierarchical relationship) or as a fellow participant (balanced equal relationship), in a manipulation of social status. We expected that participants would attribute expertise and prior knowledge to the confederate as an experimenter, and would therefore be more motivated to perform well in the experimenter condition. We also manipulated the animacy of the performances with which the participants performed: half of the performances were live, and half were recordings of the same confederate pianist. Because the confederate’s hands and arms were not visible to the participant seated across the room at a separate piano and the confederate performed the music in each duet performance, the participants did not know whether they were hearing live or recorded performances. We expected that the recorded performances, which did not permit temporal adaptation in both directions, would yield unidirectional coupling from participant to confederate (recording), while the live performances would yield the possibility of bidirectional coupling between the two pianists and thus more synchrony between performers. Finally, the auditory feedback from each pianist’s performances was presented or was removed (four levels) from the headphones of each pianist across conditions (both pianists heard the same feedback within conditions). We expected that asynchronies would worsen as feedback was systematically removed across the four conditions, with greater worsening when it was removed from the confederate’s part than the participant’s part.

## Materials and Methods

### Participants

The participants were *N* = 24 adult pianists (*M* age = 25.79 years, *SD* = 10.24) with a minimum of 8 years of piano formal instruction (*M* = 13.1, *SD* = 3.5). Twenty one of the 24 participants were right-handed, 17 were female and none had known hearing difficulties. Participants were recruited from the Montreal music community. A pre-screening test required participants to play a musical melody (described below) twice without error, and all 24 participants passed. A 21-year-old right-handed male confederate with 8 years of formal piano instruction and no known hearing difficulties performed with each participant in the duet conditions. He was instructed to limit his head and body movements across all performances.

The two social groups: those told they were performing with partners or with experimenters, were compared in terms of their age, amount of musical training, gender, familiarity with the musical piece, and whether they had formally prepared the piece for performance prior to the experiment. Comparisons are shown in **Table 1**. There were no significant differences between the groups.

**Table 1:** Participant characteristics by Social Status group.

	Experimenter		Partner				
	Mean	*SD*	Mean	*SD*	*t*	*p*	*d*
Age	24.75	(6.45)	26.83	(13.23)	-0.49	0.63	0.29
Piano training (years)	13.50	(3.06)	12.67	(4.21)	0.55	0.58	0.23

	**Frequency**	**(%)**	**Frequency**	**(%)**			

Females	9	(75.00)	8	(66.67)			
Familiarity with piece	8	(66.67)	8	(66.67)			
Formally studied piece	2	(16.67)	1	(8.33)			

### Materials and Apparatus

The pianists (the participant and the confederate) sat facing each other at two keyboards with weighted keys (Roland RD-700s), and received feedback from themselves and from their partner through Bose QuietComfort 20 Noise Canceling headphones. Piano (GM2_002, no reverberation) and metronome (GM2_232) sounds were generated by a Roland Mobile Studio Canvas Sound Module (SD-50). The FTAP program (Finney, 2001) was used to generate feedback manipulations, play the metronome and keyboard sounds, and record output from the keyboards in MIDI format on a Linux (Fedora) computer (Dell T3600).

### Musical Stimulus

**FIGURE 1:**
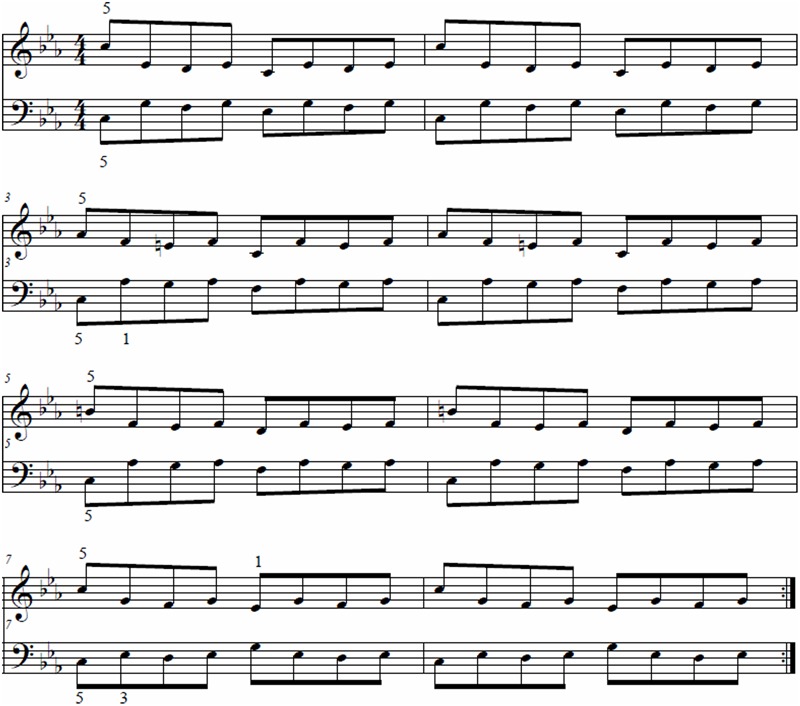
**Stimulus adapted from opening 4 bars of J. S. Bach’s Prelude in C Minor, BWV 847 (renotated in eighth notes)**.

The musical excerpt used for both the pre-test and experimental trials was the opening 4 bars (re-notated into 8 bars of eighth notes; see **Figure 1**) from J. S. Bach’s Prelude in C Minor, BWV 847. Each performance consisted of playing the excerpt three times at a tempo provided by a metronome set to one quarter-note Interonset Interval (IOI) = 225 ms. The stimulus was chosen for its rhythmically isochronous nature, as well as the equivalent difficulty between the hands. Participants were sent the sheet music prior to testing, and were asked to practice the stimulus prior to a pre-test.

### Design

The study employed a mixed design with one between-subject factor of the confederate’s Social Status (introduced as experimenter or participant) and two within-subject factors of Auditory Feedback (four levels) and Animacy of the confederate’s performances (live or pre-recorded). The four within-subject Auditory Feedback manipulations included hearing full sound (“Both present” condition), participant sound only (“Confederate-removed” condition), confederate sound only (“Participant-removed” condition), or hearing no sound (“Both-removed” condition).

#### Social Status

The participants were randomly assigned to one of two social status conditions: Half of the participants (12) were assigned to a condition in which they were told that the confederate was an experimenter in the study, and half were told the confederate was another participant, with the goal of inducing a change in the perceived social hierarchy of the participant-confederate relationship.

#### Auditory Feedback

There were four conditions of auditory feedback removal. In each condition, both the confederate and the participant heard the same auditory feedback. In the Both-present feedback condition, both participants heard feedback from both parts. In the Participant-removed condition, sound was presented from the confederate’s part only, again to both performers. In the Confederate-removed condition, sound was presented from the participant’s part only, to both performers. In the Both-removed condition, no feedback was presented to either performer. The last three conditions are referred to as auditory perturbations, during which performers were instructed to continue performing. The perturbation duration lasted for 9–12 notes. At the end of a perturbation, full auditory feedback would begin for the next 10–24 notes, after which another perturbation window could begin. The recovery period provided time for participants to return to baseline synchrony. The starting points of the perturbations were balanced across strong and weak beats and across durations within each condition.

#### Animacy

There were two conditions of the confederate’s performance *Animacy*: a live performance (an ‘animate’ partner) or a pre-recorded performance (an ‘inanimate’ partner). The confederate recorded a total of 20 recordings (both parts performed together) over the course of 4 days, and 8 (four upper part and four lower part) were selected based on their similarity to one another along the dimensions of tempo (IOI *M* = 230.85, *SD* = 8.60) and variability (CV *M* = 0.38, Range = 0.31–0.46). The confederate continued to perform on the keyboard during all trials, and the screen between the pianists prevented the participant from seeing the confederate’s hands, arms, and torso, thus removing knowledge of which trials were live or pre-recorded.

#### Blocking

The participant was randomly assigned to perform either the upper voice (using the right hand) or lower voice (using the left hand) for eight trials of the first block of the experiment, and the confederate was assigned to perform the alternative part. In the second block, the participant and confederate switched parts (and hands) for the last eight trials. Within each block, four trials contained manipulations with full auditory feedback, and four trials contained six instances each of the three auditory feedback manipulations in randomized order. Of the six auditory perturbations, two were removals of the participant’s sound only, two were of the confederate’s sound only, and two were removals of both performers’ sound. The three different perturbation conditions were presented in a counterbalanced order both across and within blocks to control for practice effects. Half of the trials were live performances of the confederate and half were pre-recorded performances, presented in a counterbalanced order across the experiment, across each block of performances that differed by assignment of participant to part, and across each sub-block of four performances with and without auditory feedback. All pre-recorded performances were also counterbalanced across the entire experiment to ensure that each participant performed with all eight pre-recordings. Thus, half of the performances were played with auditory feedback removal and half were played without; half of the performances were with a live confederate, and half with a recorded confederate; and half of the participants performed with a confederate known as an experimenter and half known as a participant.

### Procedure

Participants were given a pre-test to confirm they could play the piece three times through without error. After passing the pre-test, the confederate entered the room and participants performed the stimulus once with the confederate. The confederate was not known to any of the participants. The participant played on a keyboard facing the confederate, with the hands, arms, and torso of the confederate occluded from view by a screen, in order to prevent visual cues of the confederate’s movements and to reduce the possibility of knowing whether the performance was live or a recording. The confederate’s head and shoulders were still visible to the participants.

The participant and confederate then continued the 16 experimental trials, in which each performance consisted of an initial metronome cue of four ticks presented at a quarter note IOI of 550 ms. Participants were instructed to stop playing at the sound of a cymbal, which occurred between 1 and 5 notes after the end of the third repetition of the musical stimulus. Any trials on which the participant played too fast or too slow relative to the metronome cue, or performed the beginning of the trial with pitch errors (keypresses that generated pitches that differed from the information indicated in the musical score) were stopped at the start of the trial, and restarted up to three times.

After the completion of all duet performances, participants completed a post-test questionnaire on social aspects. In addition to the behavioral aspects of the design, results from the post-test questionnaire were examined to determine whether the social interaction of playing with a partner influenced the asynchrony of the pair. Six measures of the relationship with the confederate were tested, each on a 7-point Likert scale: how *likeable* the confederate was, how *stressful*, how *smooth*, and how *pleasant* the participant found interacting with the confederate to be, and how *connected* they felt to the confederate. There was also a measure of how *successful* participants thought their synchronization was, also measured on a 7-point Likert scale.

### Data Analyses

Both the participants’ and confederate’s performances were examined first for pitch errors. Any perturbation window within which a pitch error occurred by either performer was excluded from analysis; this resulted in the exclusion of 15.2% of trials. Pitch errors occurred less often in the primary (upper-frequency) voice (5.0%) than in the secondary (lower-frequency) voice (10.2%), consistent with previous studies of errors in piano performance (Palmer and van de Sande, 1993, 1995). Due to the differences in error rates, analyses were conducted collapsed across voices (the assignment of voice was a within-subjects variable). The dependent variables of IOI and absolute asynchrony (confederate [live or recorded] – participant), based on tone onsets, were then computed. Asynchronies greater than 3 standard deviations (1.4% of all asynchronies) were excluded from analysis. Signed asynchronies were evaluated for potential Social status effects on leadership. Finally, mean absolute asynchronies and IOIs were computed within each perturbation window and analyses were conducted on the mean values across trials by the factors of Animacy, Feedback, and Social Status.

Analyses were conducted in R (3.3.1) with the afex package (Singmann et al., 2016) used to calculate the ANOVAs. The Lsmeans package (Lenth, 2016) was used for follow-up testing using corrected degrees of freedom for statistical violations (Kenward–Rogers method).

## Results

### Confederate’s Live and Pre-recorded Performances

First, the confederate’s entire pre-recorded and live performances were compared on dimensions of tempo (measured by mean interonset interval, IOI) and variability (measured by standard deviation of IOIs, SD), to confirm that participants heard performances of equivalent temporal variability in the two Animacy conditions. The confederate’s live performances varied across participant; since there were 24 participants and four live trials each, this resulted in 92 live confederate trials compared with four pre-recorded performances. A bootstrap method was applied to the live confederate trials for comparison with the pre-recorded trials. 1000 subsamples of four trials were sampled with replacement from the set of 92 live confederate’s trials. The mean IOI was recalculated for each subsample, to provide an overall bootstrap estimate for comparison with the confederate’s pre-recorded performance IOIs. This procedure was undertaken for live performances when the confederate was introduced as experimenter and as partner to the participant. The bootstrap means and standard deviations are displayed with the observed pre-recorded counterparts in **Table 2**, which suggested no observable differences between the means or standard deviations for the two sets of performances.

**Table 2:** Timing characteristics of confederate’s live and recorded performances by social status group (after outliers due to participants’ pitch errors removed).

	Experimenter				Partner			
	Live	Recorded	*t*	*p*	Live	Recorded	*t*	*p*
Mean IOI (ms)	231.59	230.33	0.11	0.92	233.67	230.54	0.28	0.79
SD of IOI (ms)	15.48	17.11	1.06	0.53	15.56	16.54	1.10	0.56

### Effects of Perturbations on Interonset Intervals

**FIGURE 2:**
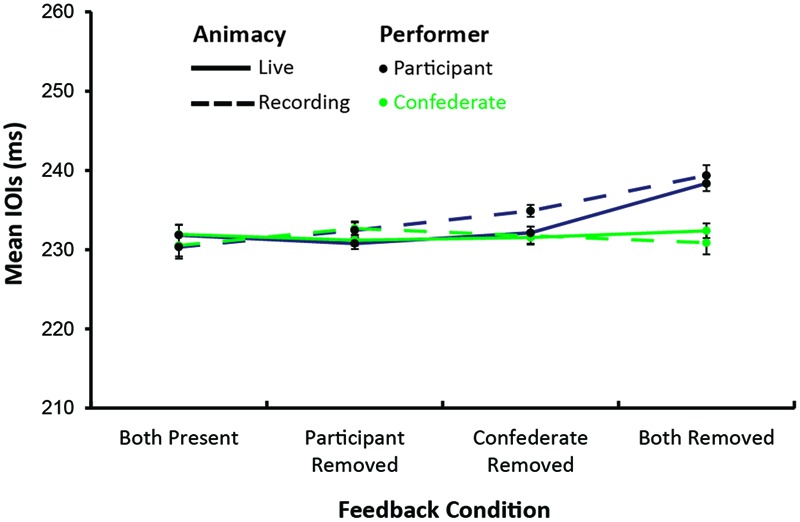
**Mean interonset intervals (ms) for participant (black) and confederate (green) in live (solid) and recorded (dashed) performances by auditory feedback condition**.

Next, we compared the confederate’s mean IOIs within the perturbation windows. The confederate’s mean IOI values for each perturbation window are shown by condition in **Figure 2**. An analysis of variance on those values by Social Status, Feedback Condition, and Animacy indicated no significant main effects or interactions. As **Figure 2** suggests, the Confederate’s tempo remained stable across conditions.

**Figure 2** also shows the participants’ mean IOIs within the perturbation windows by condition. The same analysis of variance on those values indicated a significant effect of Feedback condition, *F*(3,66) = 18.43, *MSE* = 32.84, $\eta_{G}^{2}$ = 0.20, *p* < 0.001, and the interaction of Feedback with Animacy approached significance, *F*(3,66) = 2.53, *MSE* = 15.48, $\eta_{G}^{2}$ = 0.02, *p* = 0.06. As shown in **Figure 2**, participants’ performances slowed most when auditory feedback from both parts was removed; *post hoc* comparisons indicated the Both-removed condition was slower than all other conditions (Tukey’s HSD = 6.53, *p* < 0.001). The removal of sound slowed participants’ performance slightly less when the confederate was introduced as an experimenter, but the difference did not reach significance.

### Asynchronies across Entire Performance

**FIGURE 3:**
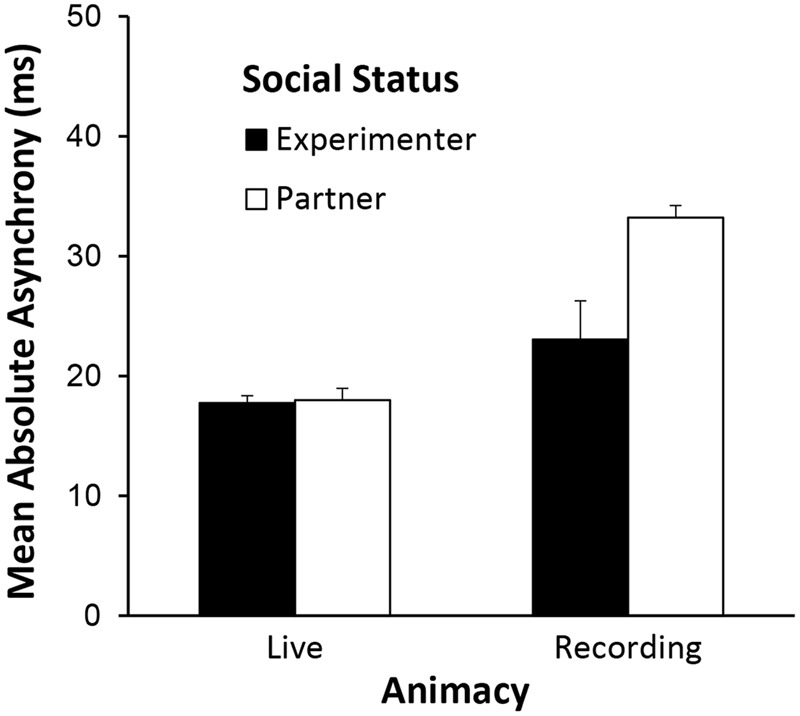
**Mean absolute asynchronies (in ms) in entire baseline performances (“sound present” auditory feedback condition) for live and recorded performances by social status of confederate (experimenter or partner)**.

The absolute asynchronies between participant and confederate were first evaluated across the entire performance of the Full Sound condition, to confirm the representativeness of the patterns of behavior measured in the perturbation windows. **Figure 3** shows the mean absolute asynchrony (participant and confederate’s tone onsets, in ms) for all simultaneities as notated in the musical score, by Social Status and Animacy. The mean asynchronies indicated significant effects of Animacy, *F*(1,22) = 19.87, *MSE* = 63.93, $\eta_{G}^{2}$ = 0.26, *p* < 0.001, and a significant interaction of Social Status with Animacy, *F*(1,22) = 4.62, *MSE* = 63.93, $\eta_{G}^{2}$ = 0.08, *p* = 0.04. As shown in **Figure 3**, asynchronies were larger for pre-recorded than for live performances, as expected; this contrast was larger when the confederate was introduced as a partner [live – recording: *t*(22) = -4.67, *p* < 0.001] than when he was introduced as an experimenter, *t*(22) = -1.63, *p* = 0.12. The main effect of Social Status approached significance, *F*(1,22) = 3.31, *MSE* = 98.06, $\eta_{G}^{2}$ = 0.08, *p* = 0.08; asynchronies tended to be larger when the confederate was introduced as a partner than as an experimenter.

To test the possibility that the participants’ response to the social status of the confederate was to use a strategy of following (lagging) the confederate when introduced as experimenter versus participant, we also measured the signed asynchronies across the entire live performances, defined as participant’s tone onsets minus confederate’s tone onsets. The mean signed asynchronies in the Both-present condition were equivalent when the confederate was introduced as experimenter (*M* = 4.96 ms), and as partner [*M* = 5.41 ms; *t*(22) = 0.16, *p* = 0.88], indicating that the participants did not alter any strategy to lag or lead the confederate in response to how the confederate was introduced across the live performances.

### Effects of Perturbations on Asynchronies

**FIGURE 4:**
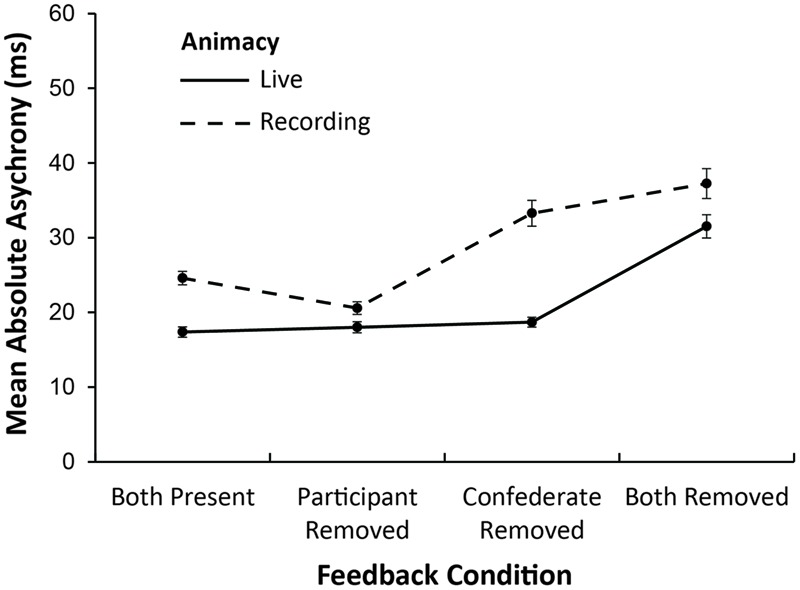
**Mean absolute asynchronies (ms) for live and recorded performances by auditory feedback condition**.

The absolute asynchronies during the perturbation windows were tested next for the effects of Social Status, Feedback condition, and Animacy. **Figure 4** shows the mean values. Main effects of Feedback condition, *F*(1,22) = 60.14, *MSE* = 36.62, $\eta_{G}^{2}$ = 0.46, *p* < 0.001, and of Animacy, *F*(1,22) = 57.68, *MSE* = 47.07, $\eta_{G}^{2}$ = 0.26, *p* < 0.001, indicated that asynchronies were larger when performances were pre-recorded than when they were live, as expected. In addition, asynchronies increased as feedback was removed, with larger asynchronies in the Both-removed condition than in the Both-present condition (Tukey contrasts), *t*(66) = 10.85, *p* < 0.001, the Participant-removed condition, *t*(66) = 12.22, *p* < 0.001, and the Confederate-removed condition, *t*(66) = 8.40, *p* < 0.001. The Confederate-removed condition generated significantly larger asynchronies than the Both-present condition, *t*(66) = 4.05, *p* < 0.001, and the Participant-removed condition, *t*(66) = 5.41, *p* < 0.001, and significantly smaller asynchronies than the Both-removed condition, *t*(66) = -6.80, *p* < 0.001. The main effect of Social Status approached significance, *F*(1,22) = 3.10, *MSE* = 87.07, $\eta_{G}^{2}$ = 0.03, *p* = 0.09, with slightly larger asynchronies when the confederate was introduced as a partner (*M* = 26.34 ms) than as an experimenter (*M* = 23.97 ms).

There was also a significant interaction of Feedback condition with Animacy on the asynchronies, *F*(3,66) = 8.63, *MSE* = 36.09, $\eta_{G}^{2}$ = 0.11, *p* < 0.001. As shown in **Figure 4**, removal of the participant’s feedback decreased the asynchronies in the recorded performances such that they did not differ from the asynchronies in the live performances. Live performances generated uniformly smaller asynchronies than pre-recorded performances for Both-present (Tukey contrast), *t*(86.7) = -4.01, *p* < 0.001, Confederate-removed condition, *t*(86.7) = -8.11, *p* < 0.001, and for Both-removed condition, *t*(86.7) = -3.18, *p* < 0.01.

The increased asynchronies in the no-sound condition coincided with the participant’s slower tempo (as shown in **Figure 2**), suggesting that this was the most difficult condition. To confirm that the asynchrony effects in the Both-removed condition were not simply due to tempo effects, the analyses were recomputed for windowed asynchrony values divided by the previous IOI (IOI was based on participant in the first analysis, and on mean of participant and confederate in a second analysis). The ANOVAs reported above were repeated on the adjusted asynchronies; the main effects and interactions were unchanged from those reported, suggesting that the difficulty due to feedback removal affected both coordination and tempo.

### Effects of Social Status on Perceived Interaction

Participants’ responses to questions about the social interaction were compared for the two Social Status groups who were introduced to the confederate as experimenter and as partner; each question was answered on a scale of 1–7. **Table 3** shows the mean values for responses by each group. As shown in **Table 3**, participants who were introduced to the confederate as an experimenter judged their interaction to be significantly smoother and more pleasant overall than those who were introduced to him as a partner. Interestingly, this difference is in the same direction as the asynchrony values, which were slightly larger (3 ms) for the partner-introduced than the experimenter-introduced performances (although the difference did not reach significance).

**Table 3:** Mean responses to social interaction questionnaire by confederate’s social status.

	Social status					
	Experimenter		Partner		Mann–Whitney	
Social interaction	Mean	*SD*	Mean	*SD*	*U*	*p*
Likeable partner	5.92	(0.90)	5.25	(1.29)	93.0	0.221
Stressful interaction	1.67	(0.78)	2.58	(1.44)	45.0	0.108
**Smooth interaction**	**5.42**	**(1.24)**	**3.92**	**(1.38)**	**113.0**	**0.017**
**Pleasant interaction**	**5.67**	**(1.23)**	**4.92**	**(0.79)**	**105.0**	**0.048**
Unconnected with partner	3.42	(1.73)	4.08	(1.78)	57.0	0.393

In addition, participants were asked whether they successfully synchronized with their partner, using a 7-point scale (1 = Not at all, 7 = Very much so). Participants who were introduced to the confederate as an experimenter judged their synchronization as more successful (mean score = 5.92) than those who were introduced as partner (*M* = 3.75, Mann–Whitney *U* = 123.5, *p* = 0.003). Thus, both perceived social interaction and perceived synchronization success were influenced by the social status of the partner manipulation.

## Discussion

This study identified three major factors that influence the balance in temporal coordination among performing musicians. We measured duet performances of pianists each of whom performed with both live and recorded performances by the same confederate pianist. To our knowledge, this was the first study to compare animate (live) and inanimate (recorded) and social imbalance conditions in the same experiment, allowing a comparison of bidirectional and unidirectional coupling effects by the same performer. Consistently larger asynchronies were observed in performances of recorded than live performances across all conditions, consistent with the hypothesis that performers used bidirectional coupling during live performances and unidirectional coupling when playing with recorded performances (Riley et al., 2011). This finding held when the timing characteristics (tempo mean and variability) of the confederate’s performances were equivalent across live/recorded performances, and across the removal of auditory feedback from participant and confederate parts.

The study also investigated the role of the partner’s social status on temporal coordination. The knowledge that the participants believed the confederate had about the task created a balanced (equal) partner relationship of participant and confederate for half of the participants, and an unbalanced (hierarchical) relationship with the “experimenter” for the other half. Slightly larger asynchronies, which reflect more instability, were observed for participants who performed with “partners” than with “experimenters.” This effect was significant only when participants played with recordings (**Figure 3**). The weak effect is perhaps not surprising for experienced musicians, as they rely on an ability to perform in imbalanced relationships (conductor-orchestra) as well as with musicians of unequal experience.

Larger effects of social status were observed in the participants’ judgments of perceived synchrony. Ratings given by participants in the “experimenter” confederate group were significantly higher than the “partner” group for the question of how successful they perceived their synchronization to be. Although the social imbalance manipulation did not create large instabilities in the observed piano keystroke asynchronies, it did create differences in participants’ perceived success in synchrony. One possibility is that the label “experimenter” heightened performers’ awareness or attunement to the temporal instability. The notion of temporal attunement has been applied to music explicitly to capture listeners’ anticipatory behavior for when rhythmic events will occur (Drake et al., 2000). Thus, performers may have been more temporally attuned to the confederate when the social manipulation made the confederate’s role more important. Another possibility for the disparity between observed and perceived synchrony was a desire to please the experimenter; participants did give higher ratings for the smoothness of their interaction with the confederate, and how pleasant they found it (**Table 3**), when the confederate was introduced as experimenter. They did not, however, rate the confederate more likeable when introduced as experimenter than partner. Thus, the manipulation of social balance between partners seemed to change their perception of their social interaction more than their degree of temporal coordination.

Removal of auditory feedback from both pianists’ headphones also created an imbalance between the duet pianists. As expected, asynchronies were largest when feedback from both parts was removed. In addition, feedback removal from the confederate’s part caused larger asynchronies than feedback removal from the participant’s part in the live performances, consistent with previous findings (Goebl and Palmer, 2009). Feedback removal of the participant’s or confederate’s parts did not change synchronization with recordings, presumably because the inanimate recordings permit only unidirectional coupling.

In sum, temporal coordination in joint music performance provides an excellent testing ground for dynamical systems principles of coupling that facilitate the maintenance of a stable phase relationship. The current study has demonstrated how auditory feedback provides information to guide that coupling, and how the animacy of the performance (live or recorded) alters the type of coupling (bidirectional or unidirectional). The findings are also consistent with the dynamical model’s assumption that bidirectional coupling between partners, available in live performance, yields an optimal form of coordination, compared with unidirectional coupling, such as what arises when a performer plays with a recording. The effects of social status on temporal coordination and perceived synchrony are consistent with previous findings that temporal synchrony and perceived affiliation are correlated in tapping tasks (Hove and Risen, 2009). The unresponsive partner: a performer who does not react (“why aren’t you listening to me?”, cried the soloist to the accompanist), requires the other member to do all the adapting for the pair to stay together.

## Ethics Statement

The McGill University Research Ethics Board reviewed and approved the study. Both oral and written (signed) consent was obtained from all participants prior to their participation in the experiment.

## Author Contributions

AD: contributed to the conception, design, data acquisition, analysis, interpretation, drafting, and revising. DC: contributed to the conception, design, data acquisition, analysis of data, drafting. MW: contributed to the conception, design, revising of the work. CP: contributed to the conception, design, interpretation, drafting, revising.

## Conflict of Interest Statement

The authors declare that the research was conducted in the absence of any commercial or financial relationships that could be construed as a potential conflict of interest.
